# Targeting L-Lactate Metabolism to Overcome Resistance to Immune Therapy of Melanoma and Other Tumor Entities

**DOI:** 10.1155/2019/2084195

**Published:** 2019-11-03

**Authors:** René G. Feichtinger, Roland Lang

**Affiliations:** ^1^Department of Pediatrics, University Hospital Salzburg, Paracelsus Medical University, Salzburg 5020, Austria; ^2^Department of Dermatology and Allergology, University Hospital Salzburg, Paracelsus Medical University, Salzburg 5020, Austria

## Abstract

Although immunotherapy plays a significant role in tumor therapy, its efficacy is impaired by an immunosuppressive tumor microenvironment. A molecule that contributes to the protumor microenvironment is the metabolic product lactate. Lactate is produced in large amounts by cancer cells in response to either hypoxia or pseudohypoxia, and its presence in excess alters the normal functioning of immune cells. A key enzyme involved in lactate metabolism is lactate dehydrogenase (LDH). Elevated baseline LDH serum levels are associated with poor outcomes of current anticancer (immune) therapies, especially in patients with melanoma. Therefore, targeting LDH and other molecules involved in lactate metabolism might improve the efficacy of immune therapies. This review summarizes current knowledge about lactate metabolism and its role in the tumor microenvironment. Based on that information, we develop a rationale for deploying drugs that target lactate metabolism in combination with immune checkpoint inhibitors to overcome lactate-mediated immune escape of tumor cells.

## 1. Introduction

Long regarded as merely a metabolic waste product, there is now growing evidence that L-lactate produced in excess by cancer cells favors tumor growth and metastasis. L-Lactate exerts this tumorigenic effect, at least in part, by disrupting the normal antitumor function of certain immune cells to create an immunosuppressive tumor microenvironment. This has important therapeutic implications because the localized immunosuppression blunts the efficacy of anticancer immunotherapies. Thus, in principle, targeting lactate metabolism could be a strategy to bolster the effectiveness of cancer therapies and improve patient outcomes. Before delving into these therapeutic possibilities, we begin with an overview of lactate metabolism, especially as it relates to energy production in cancer cells.

## 2. L-Lactate Biochemistry, Sources, and Transport

**Figure 1:**
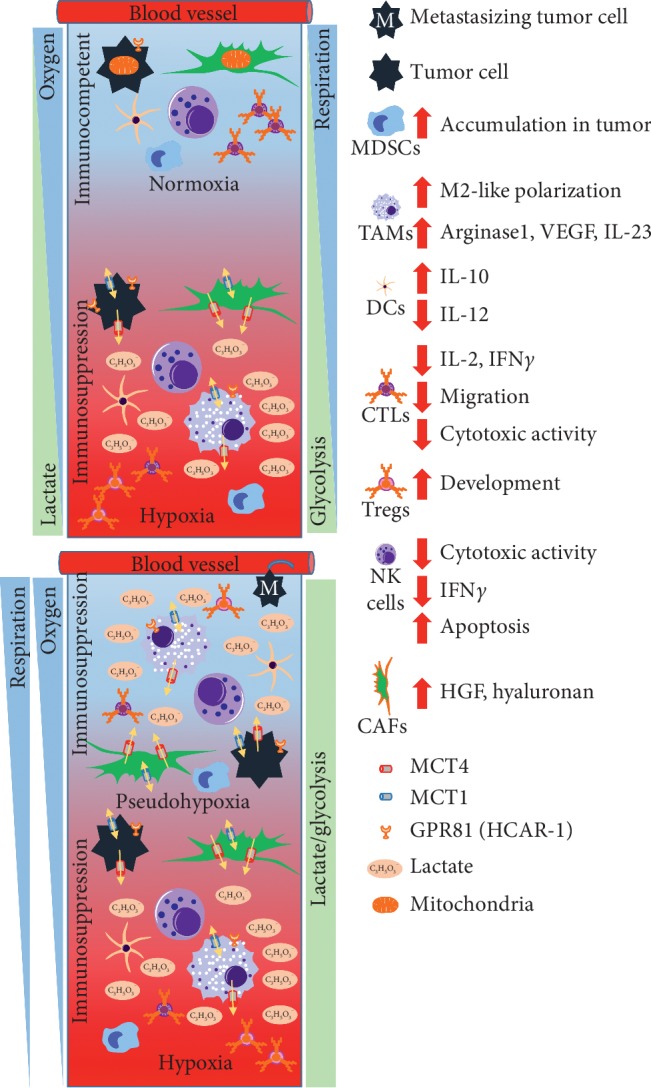
Different oxygen conditions determine the direction of the immune response in the tumor microenvironment. With increasing distance of tumor cells from blood vessels, the oxygen concentration drops. The tumor is not able to respire but instead uses primarily glycolysis for energy production with concomitant production of lactate, which in turn generates an immunosuppressive microenvironment that promotes tumor growth and metastasis (upper panel). Genetic alterations and high levels of lactate causing HIF-1*α* stabilization are responsible for the glycolytic switch. Tumors use glycolysis even if sufficient oxygen for respiration is present and express hypoxia-related genes and proteins, a state referred to as pseudohypoxia (lower panel). Mitochondria are not shown under hypoxic conditions. This represents a deficiency of OXPHOS, which can be caused by several mechanisms and not just loss of mitochondria. Cellular lactate transport is mainly executed by MCT1 (influx/efflux) and MCT4 (efflux). GPR81 is a G-protein-coupled receptor which senses extracellular levels of lactate. Increased extracellular lactate levels promote escape from immune surveillance of cancer cells, mostly through decreased cytotoxic activity of CTLs and NK cells. Furthermore, lactate induces the accumulation of MDSCs and promotes M2-like polarization and the development of tolerogenic DCs and Tregs. Secreted lactate also not only drives CAFs to produce hepatocyte growth factor, which can attenuate the activity of DCs and CTLs and promote the induction of Tregs, but also increases hyaluronan, which has been associated with cancer progression. Arrows pointing upwards indicate an increase and arrows pointing downwards a decrease. MDSCs: myeloid-derived suppressor cells; TAMs: tumor-associated macrophages; DCs: dendritic cells; CTLs: cytotoxic T lymphocytes; Tregs: regulatory T cells; NK cells: natural killer cells: CAFs: cancer-associated fibroblasts; MCT4: monocarboxylate transporter 4; MCT1: monocarboxylate transporter 1; GPR81: G-protein-coupled receptor 81; HGF: hepatocyte growth factor; VEGF: vascular endothelial growth factor.

Lactate (2-hydroxypropanoate) is a hydroxycarboxylic acid. Two stereoisomers exist, L-lactate and D-lactate. L-Lactate is the predominant enantiomer in the human body [1]. L-Lactate is either produced or removed by a reversible oxidoreduction reaction catalyzed by the enzyme L-lactate dehydrogenase (LDH). Pyruvate is reduced to L-lactate, while reduced nicotinamide adenine dinucleotide (NADH) is oxidized to NAD^+^ [2]. High levels of the LDHA isoform are found in muscles and tumors [3]. The two main sources of L-lactate in humans are pyruvate and alanine [4]. L-Lactate is the end-product of glycolysis and the pentose phosphate pathway [5]. Oxidation of L-lactate into pyruvate by LDH in the cytosol is the first step in L-lactate clearance. Lactate metabolism is a highly dynamic and tissue-specific process [6]. L-Lactate transport is mainly executed by monocarboxylate transporters (MCT1, MCT2, and MCT4) (Figure 1). MCT4 is responsible for excretion, whereas MCT1 and MCT2 work in both directions [7, 8]. In addition, two sodium-coupled monocarboxylate transporters, SMCT1 (SLC5A8) and SMCT2 (SLC5A12), mediate the cellular uptake of L-lactate [9–12]. While certain cell types excrete L-lactate, other cell types preferentially take it up, e.g., neurons and glial cells, respectively [6]. The same is true of tumor cells, tumor stem cells, tumor-associated fibroblasts, and immune cells, which provides the basis for the formation of lactate-rich tumor niches and microenvironments that are highly inimical to therapy. Moreover, it has also been proposed that lactate facilitates metastasis via creation of a microenvironment toxic to normal cells by stimulating tissue lysis [13, 14].

## 3. The Warburg Effect

The Warburg effect describes the phenomenon, wherein cancer cells generate energy predominantly via glycolysis even if sufficient oxygen for respiration is present (Figure 1). But why would tumors use inefficient glycolysis instead of oxidative phosphorylation (OXPHOS) for energy production? There are several reasons which may explain this reprogramming of ATP generation.

In normal cells, one molecule of glucose produces 38 molecules of ATP during complete oxidation in mitochondria. In cancer cells, pyruvate oxidation is downregulated and replaced by lactate production, catalyzed by LDH, without ATP generation. Thus, in tumor cells, one molecule of glucose produces only two molecules of ATP [15–17]. However, aerobic glycolysis might not be as inefficient as often reported. The production of L-lactate from glucose occurs 10–100 times faster than the complete oxidation in mitochondria and the amount of ATP production is similar per unit of time [18]. The Warburg effect has been proposed to be an adaptive mechanism to support the biosynthetic requirements of uncontrolled proliferation. Glucose serves as a carbon source for anabolic processes. The excess carbon is diverted into branching pathways emanating from glycolysis and is used for the generation of building blocks such as nucleotides, lipids, and proteins [7, 16, 19, 20]. Another theory proposes that tumors shut down OXPHOS to reduce the damage caused by reactive oxygen species (ROS) while maintaining a level necessary for signaling, e.g., especially important for chromatin metabolism [20].

## 4. Other Models

In addition to the classic Warburg hypothesis, other models have been proposed. The two primary ones are the reverse Warburg effect and the lactate shuttle hypothesis (several additional models are more or less variations of these two hypotheses). An important feature of these two models is that they take into consideration cell-cell interactions, tumor microenvironment, and compartmentalization.

In 2009, a novel “two-compartment metabolic coupling” model, also named “the reverse Warburg effect,” was proposed [21, 22]. In this model, epithelial cancer cells induce the Warburg effect (aerobic glycolysis) in neighboring stromal fibroblasts. Cancer-associated fibroblasts (CAFs) then undergo myofibroblastic differentiation and secrete lactate and pyruvate. Epithelial tumor cells are able to take up these energy-rich metabolites and use them in the mitochondrial tricarboxylic acid (TCA) cycle, thereby promoting efficient energy production (i.e., ATP generation via OXPHOS) [22].

The *intra*cellular lactate shuttle hypothesis posits that lactate formed during glycolysis can be continuously used as an energy source within mitochondria of the same cell [23]. The *inter*cellular or cell-cell lactate shuttle hypothesis proposes that lactate generated and exported from one cell can be taken up and utilized by another cell. The latter mechanism was described for neurons and astrocytes [24]. Several articles report that lactate can reach mitochondria via diffusion. LDH in the mitochondrial intermembrane space (IMS) generates NADH used by malate dehydrogenase, which converts oxaloacetate to malate. The malate-*α*-ketoglutarate (*α*-KG) antiporter (SLC25A11) transports malate into the mitochondrial matrix in exchange for *α*-KG that is transported to the IMS, where it is metabolized to glutamate by the enzyme aspartate aminotransferase (AAT). In addition, oxaloacetate is generated from aspartate. The aspartate in the IMS comes from the glutamate aspartate antiporter (SLC25A12 and SLC25A13). The glutamate in the matrix is metabolized to aspartate and the oxaloacetate to *α*-KG by AAT [23, 24].

## 5. Role of Hypoxia

A major player in the glycolytic response to hypoxia is the transcription factor hypoxia-inducible factor-1 *α* (HIF-1*α*) [25]. Following hypoxia-induced stabilization, HIF-1*α* mediates a pleiotropic reaction to hypoxia by inducing a plethora of genes, including glucose transporters, angiogenic growth factors (e.g., vascular endothelial growth factor (VEGF)), hexokinase II [26], and hematopoietic factors (e.g., transferrin and erythropoietin) [27]. Radioresistance, immune escape, and secretion of VEGF were reported to be linked to L-lactate accumulation [28–30]. Not surprisingly, MCTs are regulated by hypoxia and/or HIF-1*α* [31, 32]. Carbonic anhydrase IX (CAIX) is overexpressed in VHL-mutated clear renal cell carcinomas and hypoxic solid tumors [33, 34]. This enzyme catalyzes the reversible hydration of carbon monoxide and is thus involved in regulation of intracellular pH. CAIX is induced by HIF-1*α* [34]. Importantly, CAIX is considered to be a very reliable marker of hypoxic areas in tissue, whereas HIF-1*α* is not [35]. Hypoxia might not be important for melanomas. Although numerous articles describe changes of melanoma metabolism and behavior under hypoxic conditions, hypoxia in melanoma might not be present *in vivo*. CAIX is not expressed in the vast majority of melanocytic tumors although when it is expressed it is associated with worse overall survival (OS) [36–38]. Xu and colleagues likewise concluded that melanomas are not under hypoxic stress [39]. Although HIF-1*α* is induced by low oxygen, many other pathways can regulate HIF-1*α* in an oxygen-independent manner. The high HIF-1*α* expression observed in melanomas might be linked to increased lactate production. In other words, lactate may stimulate HIF expression independently of hypoxia [40–42]. In addition, the majority of the melanomas studied showed high OXPHOS enzyme expression, which suggests that they are OXPHOS competent. This is consistent with previous studies reporting that melanomas utilize OXPHOS in addition to glycolysis [39]. Therefore, functioning mitochondria in melanomas might be needed for oxidation of lactate produced by glycolysis.

A functioning OXPHOS system only makes sense if oxygen is present. Therefore, the majority of melanomas may be regarded as tumors that do not follow the classic Warburg rules. Several oxygen-independent pathways that regulate HIF-1*α* were identified in melanomas. Under normoxic conditions, HIF-1*α* can be stabilized by various growth factors, cytokines and oncogenes, as shown for BRAFV600E in melanoma [43]. HIF-1*α* was also identified as a microphthalmia-associated transcription factor (MITF) target [43–45]. Many factors important for neoangiogenesis are hypoxia-independent in melanomas [43]. A significant increase of LDHA expression was present in all melanomas. In addition, MCT4 was increased in single cells and areas of the melanomas, suggesting that shuttling of lactate does indeed occur [36]. However, the lactate shuttle hypothesis is still a matter of debate since the presence of LDH and MCT1 in mitochondria is questioned [46, 47]. Increased expression of SLC25A11 was reported for melanomas in a proteomics study that analyzed 61 primary melanomas [48].

## 6. L-Lactate as a Biomarker in Melanoma and Other Neoplasms

As early as 1954, increased levels of LDH were detected in serum of melanoma patients [49]. Baseline serum LDH has been established as an independent prognostic factor for survival and since 2009 has been included in the American Joint Committee on Cancer (AJCC) staging system [50, 51]. Elevated serum LDH is also a strong negative predictor of survival in patients with other hematologic and solid neoplasms [52]. Pretreatment LDH levels represent a clinically significant factor associated with response, progression-free survival (PFS), and OS in targeted therapy and immune checkpoint therapy with anti-CTLA-4- and/or anti-PD1-antibodies in melanoma patients [52–57]. High pretreatment LDH levels are also significantly associated with shorter PFS and OS in patients with advanced non‐small cell lung cancer treated with immune checkpoint inhibitors [58].

## 7. Lactate and the Tumor Microenvironment

Lactate has begun to be recognized as an active molecule capable of modulating the immune response. Tumor-derived lactate modulates the functionality of immune cells, contributing to the establishment of an immunosuppressive microenvironment which favors the development of tumors [59–61] (Figure 1). Inflammatory sites are characterized by an accumulation of lactate, which is partly responsible for the establishment of an acidic environment [62]. However, a recent review questions the presence of relevant lactate levels and its impact on immune cells in the tumor microenvironment [63].

### 7.1. Myeloid-Derived Suppressor Cells

Myeloid-derived suppressor cells (MDSCs) are a heterogeneous population of immature myeloid cells and play a crucial role in mediating immunosuppressive effects in the tumor microenvironment [64]. MDSCs suppress both innate and adaptive immunity by preventing the maturation of dendritic cells (DCs), suppressing natural killer (NK) cell cytotoxicity, inhibiting T cell activation, and favoring the differentiation of regulatory T cells [59, 60]. Tumor-derived lactate promotes the development of MDSCs [65]. One possible mechanism of suppression of NK cell function is through the induction of natural killer group-2 member D (NKG2D) ligands in tumor-infiltrating myeloid cells and circulating monocytes via tumor-derived LDH, which downregulates the activating NKG2D receptor on NK cells [28].

### 7.2. Tumor-Associated Macrophages

Tumor-associated macrophages (TAMs) are one of the most abundant cells in the tumor stroma and contribute to tumor progression at different levels [66]. Tumor-derived lactate drives macrophage polarization toward a tumor-promoting phenotype in mice [67], where HIF-1*α*-dependent lactate-induced expression of arginase 1 and VEGF might also contribute to immunosuppression and tumor evasion [67–69]. Similarly, lactate from human cervical cancer cell lines caused polarization of macrophages to an immunosuppressive phenotype [70]. Lactic acid secreted from tumor cells enhances IL-23 production in murine and human macrophages [71], which contributes to the development of protumor immunity [72]. Moreover, pretreatment of bone marrow-derived murine macrophages with lactic acid inhibited proliferation of CD8^+^ T cells [73]. Macrophages can sense lactate secreted from tumor cells via the G-protein-coupled receptors GPR132 (also known as G2A) and GPR81 (also known as hydroxycarboxylic acid receptor 1 (HCAR-1)) and respond with immunosuppressive activity [74, 75]. Both lactate and LDH in the tumor microenvironment can facilitate the protumor activity of TAMs [76].

### 7.3. Dendritic Cells and Monocytes

Some subsets of functionally distinct DC populations in the tumor microenvironment display a tolerogenic and immune suppressive phenotype [77]. High lactic acid concentrations in the tumor microenvironment possibly skew the differentiation of DCs to an immunosuppressive phenotype with increased production of IL-10 and loss of IL-12 [78, 79]. Furthermore, lactate inhibited the differentiation and lipopolysaccharide (LPS)‐induced maturation of human monocyte-derived DCs [80]. Lactate also delayed the expression or suppressed the production of proinflammatory cytokines like TNF-alpha and IL-6 in LPS-stimulated human monocytes [81, 82]. The presence of lactic acid rendered tumor-associated DCs tolerogenic and led to concentration-dependent inhibition of T cell proliferation [78]. Lactate also promoted the synthesis of prostaglandin E2 and upregulation of COX2 in monocytes, both of which are involved in tumor progression and the development of therapeutic resistance [83, 84].

### 7.4. T Cells

Several studies demonstrate that lactate negatively affects tumor immunosurveillance by T cells. Lactate suppressed the proliferation and function of murine and human cytotoxic T lymphocytes (CTLs) *in vitro* [85–87]. The presence of lactate in an acidic environment has been shown to selectively target p38 and c-Jun N-terminal kinase activation, resulting in inhibition of IFN-*γ* production in CTLs [88]. Impairment of IL-2- and IFN-*γ*-production by CTLs *in vitro* was observed following incubation with either externally added or tumor-derived lactic acid [86, 89]. Lactic acid also impairs the recruitment of CTLs to the tumor microenvironment by blocking their motility [90]. Notably, a significant decrease in intratumoral CTLs was associated with high circulating LDH levels in patients with diffuse-large B cell lymphoma [91]. Lactic acid also diminishes the cytotoxic activity of CTLs by lowering the intracellular amounts of perforin and granzyme B and reducing lytic granule exocytosis [86, 88].

Murine tumors with reduced lactic acid production caused by *Ldha* knockdown showed significantly slower growth rates and greater infiltration by functionally active CTLs compared to control tumors in immunocompetent mice [85]. Importantly, a lactate-rich tumor microenvironment not only impairs effector T cells via LDH but also fosters the development of regulatory T cells to promote immune evasion by tumor cells [92].

### 7.5. Natural Killer Cells and Natural Killer T Cells

NK cells are part of the innate tumor immune surveillance system, but their contribution is diminished by the presence of lactic acid in an acidic tumor microenvironment [92]. Similar to its effect on T cells, lactic acid prevented the upregulation of the nuclear factor of activated T cells (NFAT) in NK cells, resulting in decreased IFN-*γ* production [92] and reduced cytotoxic activity [65]. Blocking the lactate flux by inhibition of MCT4 enhanced the cytotoxicity of NK cells in a murine model of breast cancer [93]. Conversely, lactate-mediated acidification of the tumor microenvironment induced apoptosis of NK cells, resulting in their depletion from human colorectal liver metastases [94]. A high-lactate microenvironment is also detrimental to the proliferation, survival, and effector function of NKT cells [95], which are important mediators of overcoming immune exhaustion in the tumor microenvironment [96].

### 7.6. Other Cell Types

Cancer-associated fibroblasts (CAFs) are a dynamic component of the tumor microenvironment. These cells modulate the interaction between tumor cells and the host stromal response, and CAF-associated metabolic reprogramming can facilitate tumor progression [97]. Secreted lactate drives CAFs to produce hepatocyte growth factor [98], which can attenuate the activity of DCs and CTLs and promote the induction of regulatory T cells [99, 100]. Lactate also increases hyaluronan production in fibroblasts [101], and elevated hyaluronan levels in the tumor microenvironment have been linked to cancer progression and unfavorable outcomes [102, 103].

Endothelial cells are another cell type involved in the crosstalk with tumor cells in the tumor microenvironment [104]. Human umbilical vein endothelial cells (HUVECs) have been shown to respond to lactate with enhanced production of VEGF and upregulation of several receptor tyrosine kinases, including VEGF receptor 2, thereby promoting angiogenesis [105–107]. The phosphoinositide 3-kinase/Akt and NF-ϰB/IL-8 signaling pathways have been reported to be involved in mediating the proangiogenic activity of HUVECs [107, 108].

## 8. Possible Targets of Lactate Metabolism and Their Potential to Improve Immunotherapy Outcomes

Due to the multitude of effects of lactate in promoting immune evasion of tumors and stimulating tumor angiogenesis, targeting lactate metabolism in combination with immunotherapy is a promising approach to enhance the efficacy of immune therapies. This was recently demonstrated in a murine melanoma model, where blockage of LDHA not only increased the number of NK cells and CTLs but also augmented their cytolytic activity, resulting in reduced melanoma growth in combination with antiprogrammed cell death protein-1 (PD-1) therapy in comparison with PD-1 therapy alone [109]. In addition to LDH, there are other attractive molecules to target to interfere with lactate metabolism; these are described in detail below.

### 8.1. LDH

Although genetic disruption or silencing of LDHA was shown to inhibit tumor growth *in vitro* and *in vivo* in several studies [2, 110–112], it has been suggested that only disruption of LDHA and LDHB together can abolish the growth of tumor cell lines *in vitro* [113, 114].

Several LDH inhibitors have been tested preclinically for anticancer activity, but the majority of them have low potency and off-target effects and therefore are not suitable for clinical use [3].

Oxamate, a known LDH inhibitor for more than 60 years [115], is the most widely used substance for LDH inhibition in preclinical studies. However, due to its activity in the millimolar range, it has never been used in clinical trials [113, 116].

Quinoline-3-sulfonamides have been shown to have antitumor activity, but their clinical use is hampered by their poor bioavailability [112, 117].

A 2-amino-5-aryl pyrazine and a 2-thio-6-oxo-1,6-dihydropyrimidine were identified as potent inhibitors of human LDH, but they showed only minimal cellular activity in cancer cells [118, 119]. Modification of small molecule LDH inhibitors led to the development of the potent LDH inhibitor GNE-140, which inhibited murine B16 melanoma as well as human adenocarcinoma and pancreatic carcinoma cells *in vitro* dependent on their metabolic activity [114, 120].

Other drugs which target LDH by different mechanisms and exhibit preclinical antiproliferative activity against cancer cell lines, such as galloflavin [121, 122], FX11 [2], and *N*-hydroxyindole-2-carboxylate- [123, 124], and pyrazole-based inhibitors of LDH [125], have never been used clinically.

Recently, molecules with 1,4-triazole moieties have been reported as potent inhibitors of LDH, but they have not been tested for anticancer activity [126].

Several natural products, including the saffron derivative crocetin, have been identified as LDH inhibitors with antiproliferative activity against cancer cell lines [127].

Gossypol (also known as AT-101), derived from cotton plant seeds, is a nonselective inhibitor of LDH whose antitumor activity has been attributed to its additional capability to inhibit the antiapoptotic Bcl-2 protein family [128]. Gossypol has been tested in several phase I and phase II clinical trials in various tumor types either as a monotherapy or in combination with chemotherapy but produced negligible response rates in the majority of studies. Despite the multiple biological properties of gossypol, oral doses up to 40 mg per day were tolerated [129–134].

Oroxylin A, a bioactive flavonoid isolated from a Chinese medicinal plant, inhibited LDH and the production of lactate in human hepatocellular carcinoma cells [135]. However, the broadly reported anticancer activity of oroxylin A, including its inhibitory action on the generation of regulatory T cells in the tumor microenvironment of non‐small cell lung cancer, appears to involve multiple targets and pathways [136, 137].

A recent high-throughput screen of 1280 drugs identified vitamin C as an LDH-lowering agent, which reduced lactate production and inhibited tumor growth of breast cancer cells in a chronic stress model [138].

There are several drugs currently approved for clinical use which could potentially be repurposed as LDH inhibitors such as the antiepileptic drug stiripentol [139] or the nonsteroidal anti-inflammatory drugs (NSAIDs) diclofenac and lumiracoxib [140].

### 8.2. MCTs

As knockdown of the lactate transporters MCT1 and MCT4 resulted in suppression of breast cancer and colorectal cancer *in vitro* and *in vivo* [141, 142], targeting MCTs has also been included in therapeutic strategies. Accordingly, analogs of *α*-cyano-4-hydroxycinnamic acid [143] as well as derivatives of 7-aminocarboxycoumarins [144] have been reported as MCT1 inhibitors with remarkable antitumor activity *in vitro* and *in vivo*. While some MCT1-inhibiting small molecules have been described as immunosuppressive compounds [145], a small molecule inhibitor of MCT1, AZD3965, has shown preclinical antitumor properties in several hematological tumors [146] and small cell lung cancer [147]. The compound has also entered a phase I trial (NCT01791595) in patients with advanced solid tumors or lymphoma, but no results of this trial have been published to date.

For MCT4, diclofenac [148] and bindarit (2-[(1-benzyl-1H-indazol-3-yl)methoxy]-2-methylpropanoic acid) [149] have been reported as selective inhibitors. Because the efficacy of the MCT4 inhibitor AZ93 to block the growth of various cancer cell lines was dependent of MCT1 inhibition [8], it is likely that only concurrent inhibition of MCT1 and MCT4 can impair tumor growth, especially under hypoxic conditions. Syrosingopine was recently identified as a dual inhibitor of MCT1 and MCT4 with potential antitumor benefits *in vivo* [150]. There is evidence that lonidamine, a well-tolerated anticancer drug which is particularly effective at selectively sensitizing tumors to other therapies, might also be capable of concurrently inhibiting MCT1 and MCT4 [151, 152].

### 8.3. GPR81

GPR81 (HCAR-1) is a lactate-sensing receptor found on monocytes and other immune cells [75, 153] and also on certain cancer cells. In the latter, GPR81 activation promotes proliferation, invasion [154], chemoresistance [155], and upregulation of programmed cell death protein 1-ligand (PD-L1) [156]. Knockdown of GPR81 in mice diminished the production of IL-10 and suppressed the generation of regulatory T cells [75]. Furthermore, silencing of GPR81 in tumor cells led to reduced PD-L1 expression [156] and attenuation of growth and metastatic potential [157]. These interesting findings elevate GPR81 as another target in lactate metabolism to be included in tumor therapy approaches.

## 9. Conclusion

The Warburg effect and altered tumor metabolism have been recognized as a hallmark of cancer for nearly a century. Lactate is one of the key “oncometabolites” regulating the interaction of cancer cells with the tumor microenvironment. Since elevated serum LDH is negatively associated with clinical efficacy of anticancer (immune) therapies, targeting this enzyme or other molecules involved in lactate metabolism clearly has potential to improve patient outcomes. Although several LDH inhibitors lack selectivity and clinical efficacy in monotherapy, there may be strong potential in combining them with immunotherapy, especially in patients with high LDH levels. Possible off-target effects (either beneficial or toxic) would need to be assessed. Repurposing of approved drugs which can inhibit LDH and have been well tolerated in clinical trials could circumvent toxicity concerns. Besides inhibition of LDH, there are other key molecules involved in lactate metabolism which could be targeted to overcome resistance to immune therapy.

## Abbreviations

AAT: Aspartate aminotransferase
AJCC: American Joint Committee on Cancer
Akt: Protein kinase B
*α*-KG: *α*-Ketoglutarate
ATP: Adenosine triphosphate
Bcl-2: B cell lymphoma 2
BRAF: v-Raf murine sarcoma viral oncogene homolog B
CAF: Cancer-associated fibroblast
CAIX: Carbonic anhydrase IX
COX2: Cyclooxygenase 2
CTL: Cytotoxic T lymphocyte
CTLA-4: Cytotoxic T-lymphocyte-associated protein 4
DC: Dendritic cell
GPR81: G-protein coupled receptor 81
GPR132 (also known as G2A): G-protein coupled receptor 132
HCAR-1: Hydroxycarboxylic acid receptor 1
HIF-1*α*: Hypoxia-inducible factor-1*α*
HUVEC: Human umbilical vein endothelial cell
IFN-*γ*: Interferon-*γ*
IMS: Intermembrane space
LDH: Lactate dehydrogenase
LPS: Lipopolysaccharide
MCT1: Monocarboxylate transporter 1
MCT2: Monocarboxylate transporter 2
MCT4: Monocarboxylate transporter 4
MDSC: Myeloid-derived suppressor cell
MITF: Microphthalmia-associated transcription factor
NADH: Nicotinamide adenine dinucleotide
NFAT: Nuclear factor of activated T cells
NF-ϰB: Nuclear factor “kappa-light-chain-enhancer” of activated B cells
NK cell: Natural killer cell
NKG2D: Natural killer group 2 member D
OS: Overall survival
OXPHOS: Oxidative phosphorylation
PD1: Programmed cell death protein 1
PFS: Progression-free survival
ROS: Reactive oxygen species
SLC25A11: Solute carrier family 25 member 11 (malate-*α*-ketoglutarate antiporter)
SLC25A12: Solute carrier family 25 member 12 (glutamate aspartate antiporter)
SLC25A13: Solute carrier family 25 member 13 (glutamate aspartate antiporter)
SMCT1 (SLC5A8): Sodium-coupled monocarboxylate transporter 1
SMCT2 (SLC5A12): Sodium-coupled monocarboxylate transporter 2
TAM: Tumor-associated macrophage
VEGF: Vascular endothelial growth factor
VHL: von Hippel Lindau.
